# Supplementary material for: Exploring the Biological Activity and Mechanism of Xenoestrogens and Phytoestrogens in Cancers: Emerging Methods and Concepts
Source: Int J Mol Sci. 2021 Aug 16;22(16):8798. doi: 10.3390/ijms22168798 (PMC8395949; doi:10.3390/ijms22168798)
Supplement: Supplementary file 1 [file ijms-22-08798-s001.zip › ijms-1305500-supplementary.pdf]

Supplementary Table S1: Additional information on the clinical trials from Table 3

Identifier	Clinical Trial Name	Cancer Type/ Prevention	Chemicals	Additional Info on Participants	Additional Results	Conclusion
NCT00597532 [226]	Effect of Soy Supplementation on Cellular Markers in Normal and Cancerous Breast Tissue: A randomized Placebo Controlled Study	Breast	25.8g soy protein powder (TG) or 25.8g milk protein (PG)	Women with early-stage breast cancer	TG: ↑ plasma isoflavones (P < 0.001) No significant changes in Ki67 (P = 0.21) or Cas3 (P = 0.35) with soy intake	Soy supplementation alters breast cancer-related gene expression. "Soy intake did not result in statistically significant changes in cell proliferation and apoptosis indices compared with the placebo group."
NCT00513916 [227, 228]	Effect of Dietary Soy on Estrogens in Breast Fluid, Blood, and Urine Samples from Healthy Women	Breast	High-soy diet = 2 servings of soy foods/day<br><br>Low-soy diet = <3 servings of soy foods/week<br><br>Serving = 177 mL soy milk, 126 g tofu, or 23 g soy nuts		TG, PR vs. PO = ↑ Ki-67 labeling index (NS)	Soy does not exert antioxidant effects, as measured by urinary isoprostane excretions<br>No increase in hyperplastic epithelial cells after soy intake in amounts consumed by Asians.<br>It is safe to consume soy foods in amounts from traditional Asian diets.<br>Need to investigate additional markers of oxidative stress.
NCT00612560 [229]	Flaxseed, Aromatase Inhibitors and Breast Tumor Characteristics (FABrC)	Breast	25 g/day ground flaxseed (FS)<br>1 mg/d anastrozole (AI)	Women newly diagnosed with incident, operable, ER+ invasive breast cancer, clinical stage II or lower	NS changes in Ki-67, caspase, and Ki-67: caspase ratio<br>All TG vs P = No effect on EXP of growth-related biomarkers or estrogen receptorβ EXP	"In this small clinical trial, we found little evidence of an interaction between flaxseed and a commonly prescribed aromatase inhibitor, anastrozole, in the modification of several breast tumor characteristics related to prognosis or in serum hormone levels."

NCT00290758 [230]	Genistein in Preventing Breast Cancer in Women at High Risk for Breast Cancer	Breast	Mixed soy isoflavone PTIG-2535 (150 mg genistein, 74 mg daidzein, 11 mg glycitein)	Healthy, non-pregnant and non-lactating women at increased risk for breast cancer, or women with a history of unilateral minimal risk breast cancer	No treatment effect on cytolotic atypia or nipple aspirate fluid parameters	Mixed soy isoflavones did not reduce breast epithelial proliferation. The study suggested a lack of efficacy for breast cancer prevention. There is also a possible adverse effect in premenopausal women. There were no significant increases of the 28 genes observed between soy-treated women and the control group
NCT01219075 [231]	Soy Isoflavones Supplementation in Treating Women at High Risk For or With Breast Cancer	Breast	Novasoy 400 (12.7% daidzein, 11.57% genistein, 3.36% glycitein)	Women diagnosed with ductal carcinoma *in situ* or invasive breast cancer		No evidence has shown that soy supplementation would have any beneficial or adverse effects on breast tissue changes or decrease in mammographic density. "MRI might be more sensitive to changes in density than mammography."
University of North Dakota School of Medicine and Health Sciences [232]	Trans-resveratrol alters mammary promoter hypermethylation in women at increased risk for breast cancer	Breast	5 or 50 mg of *Trans*-resveratrol with *P. cuspidatum*	Women with an increased breast cancer risk, a Gail risk of >1.66% of developing breast cancer in the next 5 yr, and/or personal history of a breast biopsy demonstrating atypical hyperplasia, in	High-dose vs. Low-dose *Trans*-resveratrol TG= No S effect of DNA methylation in 4 genes (p16, CCND2, APC, and RASSF-1ɑ)	"Our preliminary observations suggest a novel mechanism for the chemopreventive effect of *trans*-resveratrol in the breast of high-risk women." More *trans*-resveratrol and its glucuronide metabolite in circulation, S decrease in RASSF-1ɑ methylation. "Because of the limited sample size, our findings should be validated in a larger study."

				situ, or invasive breast cancer		
National Center of Oncology, Yerevan, Armenia (Ministry of Health Republic of Armenia Registration No.: Nr 5592-17-02-23) [233]	Efficacy and safety of curcumin in combination with paclitaxel in patients with advanced, metastatic breast cancer; A comparative, randomized, double-blind, placebo-controlled clinical trial	Breast	300 mg injectable solution of Curcumin (CUC-01)	Women with advanced and metastatic breast cancer		Curcumin in combination with paclitaxel is efficacious in treatment of advanced and metastatic breast cancer. No major safety concern, no reduction in the quality of life, and a slight reduction of fatigue when curcumin is introduced intravenously.

NCT00596895 [234]	Isoflavone in Prostate-specific Antigen Recurrent Prostate Cancer	Prostate	Soy milk containing 47 mg isoflavonoid (genistein, daidzein, and equol); men consume 141 mg of isoflavone/day	Men with rising prostate-specific antigen (PSA) after prior local therapy		Isoflavone is well-tolerated and may slow the rate of rise of serum PSA in some patients with biochemical progression after radiation therapy or radical prostatectomy “This study may lend support to the literature that nutritional supplements have biologic activity in prostate cancer and therefore, further studies with these agents in randomized clinical trials should be encouraged.”
NCT01009736 [61]	Effects of Tomato-Soy Juice on Biomarkers in Patients with Prostate Cancer Undergoing Prostatectomy	Prostate	Tomato-soy juice containing 20.6 mg total lycopene, 42.3 mg genistein, 20.7 mg daidzein, and 2 mg glycitein	Men with biopsy-proven carcinoma of the prostate who had chosen a radical prostatectomy for treatment	Urinary isoflavones daidzein, genistein, and glycitein increased in a dose-dependent manner.	“These findings provide the foundation for evaluating a well-characterized tomato-soy juice in human clinical trials to define the impact on human prostate carcinogenesis.”
NCT00255125 [235]	Role of Soy Supplementation in Prostate Cancer Development	Prostate	Soy isoflavone capsules (80 mg/d of total isoflavones, 51 mg/d aglucon units)	Men with localized prostate cancer	The genes that were down-regulated in TG vs. PG = 12 genes involved in cell cycle control (CDC27, APAF1, CCNB2, CCNG1, CCNG2, CCNC, UBE1, CUL2, CUL3, E2F4, CHEK2, ATM), and 9 genes involved in apoptosis (CD40, Bcl-2, BIRC1/NAIP, BIRC2, BIRC6, BAX, TANK, HUS1, and CASP7)	Short-term intake of soy isoflavones did not affect serum hormone levels, total cholesterol, or prostate specific antigen “Changes in serum total testosterone, free testosterone, total estrogen, estradiol, PSA, and total cholesterol in the ioflavone-treated group compared to men receiving placebo were not statistically significant.”

NCT00765479 [236]	Soy Protein in Preventing Recurrent Cancer in Patients Who Have Undergone Surgery for Stage II Prostate Cancer	Prostate	Soy protein isolate (70.5 mg of all forms of isoflavones and in aglycone equivalents, 41 mg total isoflavones, 23.8 mg of genistein, and 15.0 mg daidzein)	Men who were at risk of prostate cancer recurrence following radical prostatectomy	In 2-y, serum ferritin concentrations doubled in TG & PG ($p < 0.001$), while increased slightly in hemoglobin (TG, $p < 0.001$; PG, $p = 0.002$) and hematocrit (TG, $p = 0.015$; PG, $p = 0.002$) Body weight at baseline was associated with equol production status ($p < 0.001$)	Although soy protein supplement did not affect body weight, blood pressure, serum total cholesterol, calcium, phosphorous, and thyroid hormones, subjects with equol production may modify effects of soy on body weight and possibly blood pressure.
NCT00546039 [237]	Synthetic Genistein (BONISTEIN) in Patients Who Are Undergoing Surgery for Prostate Cancer	Prostate	30 mg Synthetic genistein	Men with localized prostate cancer scheduled to be treated by radical prostatectomy	In normal cells, NS in down-regulation of androgen receptor protein expression ($p = 0.123$) and KLK4 mRNA level ($p = 0.087$) p27Kip1 expression was increased in the nuclear compartment Genistein may have an inhibitory effect on androgen-related biomarkers	"Genistein intervention modulated the expression of several biomarkers which may be related to prostate cancer prediction and progression. The present study supports genistein as a chemopreventive agent in prostate cancer. Further investigation is warranted in larger and longer-duration studies."
NCT02724618 [238]	Nanocurcumin for Prostate Cancer Patients Undergoing Radiotherapy (RT)	Prostate	120 mg/day oral nanocurcumin	Patients with histologically confirmed adenocarcinoma of the prostate and the	Both TG and PG patients experienced radiation-induced proctitis ($p = 0.313$) No significant differences were	"This randomized controlled trial was underpowered to indicate the efficacy of nanocurcumin in this clinical setting but could provide a considerable new translational insight to bridge the gap

				performance status of 0-2 who were candidates for 3D conformal or intensity modulated radiotherapy	found between TG and PG with regard to ≥ grade II proctitis, cystitis (≥ grade I/II), and duration of radiation toxicities.	between the laboratory and clinical practice.”
NCT02138955 [239]	A Phase IB Dose Escalation Study of Lipocurc in Patients With Cancer	Prostate, Colon	(1E6E)-1,7-bis (4-hydroxy-3-methoxyphenyl)-1,6-heptadiene-3,5-dione with 20 mL liposomal suspension and curcumin concentration of 6.0 ± 1.5 mg/mL	Men and women ≥ 18 years with metastatic tumors	Stable curcumin plasma concentrations during infusion followed by rapid declines to undetectable levels after the infusion. 34 of 143 adverse events experienced by 30 patients were possibly related to the study treatment	100 - 300 mg/m² of infused liposomal curcumin over 8 h a dose was generally well tolerated<br>There is a possibility that liposomal curcumin as an anti-cancer agent should be combined with other chemotherapies
NCT01917890 [240]	Radiosensitizing and Radioprotective Effects of Curcumin in Prostate Cancer	Prostate	Total 3 g/day of curcumin	Men with prostate cancer undergoing radiotherapy		Curcumin is used as an antioxidant agent and does not compromise the therapeutic efficacy of radiotherapy
NCT00118846 [241]	Women’s Isoflavone Soy Health (WISH) Trial	Endometrial	25 g soy protein containing genistein 52 mg aglycon equivalents (88 mg total), daidzein 36 mg aglycon equivalents (61 mg	Postmenopausal women	↑ mean plasma concentrations of genistein, daidzein, and glycitein in TG ($p < 0.001$)<br>9 out of 121 participants in TG underwent an	“We observed that three-year ISP supplementation had no effect on endometrial thickness or on rates of endometrial hyperplasia and cancer in postmenopausal women.”

			total), and glycitein 3 mg aglycon equivalents (5 mg total)		endometrial biopsy, and 100% of cases had benign results	
NCT02017353 [242]	Effect of Curcumin Addition to Standard Treatment on Tumour-induced Inflammation in Endometrial Carcinoma	Endometrial	2 g/day of 500mg Curcumin Phytosome (CP)	Patients with histologically confirmed endometrial carcinoma and no life-threatening metastases	In TG, NS changes in soluble inflammatory biomarker levels, frequencies of other immune cell types, T cell activation, and COX-2 expression ↓ frequency of HLA-DR expressing leukocytes and ↓ HLA-ABC EXP level in TG ($p < 0.05$) No significant changes in COX-2 expression in PBMC, B cells, NK cells and T cells upon CP supplementation. No differences observed regarding inflammatory biomarkers, frequencies of other immune cell types, T cell activation and COX-2 expression	Although the study consisted of a small number of participants, it observed minor immunomodulatory effects of curcumin supplementation in endometrial cancer patients Suggested to determine whether different supplementation regimens could induce immunological benefit in endometrial cancer The study also suggested "more research is necessary to explore whether benefits of CP can be obtained in EC (endometrial carcinoma) by different supplementation regimens."
NCT00256334 [243]	Resveratrol for Patients with Colon Cancer	Colon	20 mg trans-resveratrol (derived from	Patients with colon cancer	In TG of normal colonic mucosa, ↓ Wnt target genes (*myc*,	Both plant-derived resveratrol and freeze-dried grape powder (GP) have

		whole red grapes and *Polygonum cuspidatum* root extracts) + 120 mg of quercetin		*jun*, *TCF7*, *axinII*, and *cyclinD1*) ($p = 0.03$) Lower dose of freeze-dried grape powder (GP) = significant reduction in Wnt target gene EXP ↑ EXP of CD133 ($p < 0.05$) and LGR5 ($p < 0.005$) in cancer than in normal mucosa	effects *in vivo* on various measures of the Wnt pathway Resveratrol combined with other bioactive components present GP suppresses Wnt pathway target gene EXP "Further study of dietary supplementation with resveratrol-containing foods such as whole grapes or GP (grape powder) as a potential colon cancer preventive strategy is warranted."

TG, treatment group; PG, placebo group; PR, premenopausal women; PO, postmenopausal women; NS, non-significant; S, significant; EXP, expression
